# A Targeted In-Fusion Expression System for Recombinant Protein Production in *Bombyx mori*

**DOI:** 10.3389/fgene.2021.816075

**Published:** 2022-01-04

**Authors:** Zhiqian Li, Lang You, Qichao Zhang, Ye Yu, Anjiang Tan

**Affiliations:** ^1^ Jiangsu Key Laboratory of Sericultural Biology and Biotechnology, School of Biotechnology, Jiangsu University of Science and Technology, Zhenjiang, China; ^2^ Key Laboratory of Silkworm and Mulberry Genetic Improvement, Ministry of Agriculture, The Sericultural Research Institute, Chinese Academy of Agricultural Sciences, Zhenjiang, China; ^3^ Key Laboratory of Insect Developmental and Evolutionary Biology, CAS Center for Excellence in Molecular Plant Sciences, Shanghai Institute of Plant Physiology and Ecology, Chinese Academy of Sciences, Shanghai, China

**Keywords:** transcription activator-like effector nuclease (TALEN), silk gland, bioreactor, sericin, *Bombyx mori*

## Abstract

The domesticated silkworm, *Bombyx mori*, is an economically important insect that synthesizes large amounts of silk proteins in its silk gland to make cocoons. In recent years, germline transformation strategies advanced the bioengineering of the silk gland as an ideal bioreactor for mass production of recombinant proteins. However, the yield of exogenous proteins varied largely due to the random insertion and gene drift caused by canonical transposon-based transformation, calling for site-specific and stable expression systems. In the current study, we established a targeted in-fusion expression system by using the transcription activator-like effector nuclease (TALEN)-mediated targeted insertion to target genomic locus of sericin, one of the major silk proteins. We successfully generated chimeric Sericin1-EGFP (*Ser-2A-EGFP*) transformant, producing up to 3.1% (w/w) of EGFP protein in the cocoon shell. With this strategy, we further expressed the medically important human epidermal growth factor (hEGF) and the protein yield in both middle silk glands, and cocoon shells reached to more than 15-fold higher than the canonical *piggyBac*-based transgenesis. This natural *Sericin1* expression system provides a new strategy for producing recombinant proteins by using the silkworm silk gland as the bioreactor.

## Introduction

The lepidopteran model insect *Bombyx mori* is an important economic insect and possesses a highly specialized larval tissue, the silk gland, to synthesize and secret massive silk proteins in a few days of the late final larval instar. On average, each silkworm eats 20 g of mulberry leaves and produces about 0.5 g of pure silk protein, holding the great promise to be a cost-effective system for mass production of recombinant proteins (Ma et al., 2014). This efficient protein production capacity has been described as an important model for tissue-specific gene regulation and exogenous protein synthesis (Tomita et al., 2003; Takasu et al., 2016).

Silkworm silk proteins, which are the major components of cocoon shells, contain the insoluble fibroin protein and hydrophilic sericin protein (Iizuka et al., 2008). Fibroin protein is synthesized in the posterior silk gland (PSG), assembled in the lumen of the middle silk gland (MSG) with sericin, and then secreted into the anterior silk gland (ASG) to spin and form cocoon shells. Fibroin proteins account for 70%–80% of the total silk proteins, being composed by the heavy chain protein (FibH), light chain protein (FibL), and fibrohexamerin protein (Fhx) in a molar ratio of 6:6:1 (Inoue et al., 2005). In recent years, the transposon-based, transgenic silk gland expression system has been established to express recombinant proteins with the *FibL* promoter and its 5′-flanking sequences. The exogenous proteins were secreted into the lumen of PSG together with fibroin proteins and reached up to 0.84% of the whole cocoon shell weight (Tomita et al., 2003). Subsequently, another transgene-based expression system using the *FibH* promoter was established and promoted the recombinant protein amount to 15% (w/w) per cocoon shell (Tomita et al., 2007; Zhao et al., 2010). Most recently, a targeted expression of ampullate spidroin-1 gene with *FibH* gene replacement was developed successfully, with an unprecedented yield up to 35.2% wt/wt of cocoon shells (Xu et al., 2018). These cases suggested that fibroin genes can achieve high recombinant protein yield, however, extraction and purification of the proteins were complicated since the fibroin protein was insoluble and the exogenous proteins are tightly combined with silk fibers.

Sericin proteins weigh to nearly 20% of the total cocoon shell weight, while the extraction and purification processes from a cocoon shell are more practical since they are soluble. Sericin proteins are synthesized in the MSG and mainly consist of Sericin1 (Serl), Sericin2 (Ser2), and Sericin 3 (Ser3) proteins, in which Ser1 has the dominant expression (Takasu et al., 2007). They function as the glue protein and majorly coat and cement fibroin filaments to form the silk fibers (Xu et al., 2014; Dong et al., 2016). The transposon-based transgenic sericin expression system ectopically expressed exogenous protein with Ser1 promoter has been established and a series of modifications on the regulatory elements both at promoter and 3′ untranslated region (UTR) were performed to increase the transcriptional and translational level of exogenous proteins (Tomita et al., 2007; Iizuka et al., 2008; Wang et al., 2013; Wang, 2013). Additionally, exogenous protein yield could be increased under the mutant genetic background, which is deficient in fibroin secretion (Inoue et al., 2005). Altogether, these evidences suggested that transgenic production of exogenous proteins was largely depending on the regulatory elements, inspiring us to establish an *in situ* sericin expression system with the original regulatory sequences.

Transposon, especially the *PiggyBac*-mediated transgenesis advent the genomic era in the silkworm, however, targeted genome editing was still challengeable till the site-specific nuclease was engineered successfully (Maeder et al., 2008; Edgell, 2009; Boch, 2011; Mulepati et al., 2014). Along with the quick development and adaption of the site-specific nucleases, homing endonucleases, including ZFN (zinc-finger nuclease), TALEN (transcription activator-like effector nuclease), and CRISPR/Cas9 (clustered regularly interspaced short palindromic repeats/RNA-guided Cas9 nucleases) have been wildly applied into a large range of host organisms and cells (Zhang et al., 2014). All these nucleases created double-stranded breaks (DSBs)at the targeted genomic DNA, which trigger and utilize the endogenous DSB repair machinery especially for homologous-directed repair to introduce designed modifications or insertions. Up to now, only TALEN-mediated targeting insertion was achieved in the silkworm successfully, which may be attributed to different DSB repair pathways that were used by these engineered nucleases (Wang et al., 2013; Xu et al., 2018; Zhang et al., 2018). Here we report establishment of an *in situ Ser1* in-fusion expression system in *B. mori* by using the TALEN-mediated targeted insertion. High production of exogenous proteins of enhanced green fluorescent protein (EGFP) and the medically important human epidermal growth factor (hEGF) were successfully detected in both MSGs and cocoon shells. Compared with the transposon-based random insertion, our strategy promoted the hEGF production up to 15-fold. In conclusion, the current study established a natural *Sericin1* bioreactor system in *B. mori*, showing great potential for mass production of recombinant proteins.

## Materials and Methods

### Silkworm Strains and Cell Line

The multivoltine, nondiapausing silkworm strain, Nistari, was used for genetic transformation. Larvae were reared on fresh mulberry leaves under the standard condition at 25°C (Tan et al., 2013). Mammalian HEK293T cell was maintained in DMEM (Gibco) medium supplemented with 10% fetal bovine serum at 37°C under 5% CO_2_.

### Construction of Tanscription Activator-Like Effector Nuclease and Homologous Recombination (HR)-Mediated Donor Plasmids

Pairs of TALENs were designed and constructed by ViewSolid Biotech using Golden-Gate assembly and ligated into the VK006-06 vector, under the control of T7 *in vitro* transcriptional promoter. The activity of the TALENs was examined using an SSA assay in HEK293T cell line (Wang et al., 2013). One TALEN targeting sites located around the stop codons (C) of the *Sericin1* gene was chosen with the sequence listed as follows: 5′-TAA​GAA​TAT​CGG​TGT​TTa​ata​caa​cta​aac​acg​aCT​TGG​AGT​ATT​CCT​TGT​A-3′, with the capital letters as the TALEN recognition sites and lowercase letters as the spacers. The targeting sites were verified by amplification from the genomic DNA to exclude the single nucleotide polymorphisms. A homology-directed recombination (HDR) donor plasmid was constructed based on the pGEM-T vector. For facilitating the HR recombination, the *HR5-IE1-DsRed-SV40* cassette was cut from *pXL-IE1-DsRed* silkworm transgenic plasmid using BamHI single restriction enzyme, and subcloned into the pGEM-T easy vector (Promega) to generate pGEM-Red plain plasmid. A 1,000-bp 3′-HR arm amplified from the genomic DNA at the right flanking of the TALEN site was inserted into the pGEM-Red plasmid at the SpeI restriction enzyme site using in-fusion ClonExpress™ II One Step Cloning Kit (RA, Vazyme Biotech Co. Ltd.). The 1,000-bp left HR arm was amplified from the left of the TALEN site, which was then fused with the *EGFP* or *hEGF* expressing cassette. To achieve the sericin in-fusion expression, *EGFP-* or *hEGF-*coding sequences was inserted into the downstream of the right homologous arm using 2A self-cleavage sequence (GAG​GGC​AGA​GGA​AGT​CTT​CTA​ACA​TGC​GGT​GAC​GTG​GAG​GAG​AAT​CCC​GGC​CCT) at the SacII restriction enzyme site. To optimize the exogenous proteins expression, the *Sericin1* polyA (PA) sequence was cloned and ligated into the downstream of *EGFP* or *hEGF* sequence. In order to facilitate the integration, two TALENs targeting sequences were added to each side of the donors, to linearize the circular donor plasmids.

### Preparation of Transcription Activator-Like Effector Nuclease mRNA and Microinjection

TALEN-expressing vector under the control of the T7 *in vitro* expression promoter was linearized using the NotI restriction enzyme, purified with phenol:chloroform:isoamyl alcohol (25:24:1), and sent for *in vitro* mRNA synthesis using the mMessage mMachine T7 Ultra Kit (Life Technologies). Mixture of the TALEN mRNA (250 ng/μl) and donor plasmid (300 ng/μl) was injected into silkworm embryos at the preblastoderm stage (Li et al., 2018). The injected eggs were incubated at 25°C for 10–12 days until hatched and reared on fresh mulberry leaves. G0 moths were crossed with wild-type (WT) animals and positive G1 embryos were screened under red fluorescence.

Total RNA extraction, first strand cDNA synthesis, and quantification of mRNA total RNA was extracted from the middle silk gland of *WT*, *Ser-2A-EGFP*, *Ser-2A-hEGF*, and *Ser-T-hEGF* animals during the whole fifth instar larvae. One microgram of the purified RNA was used for the cDNA synthesis using the ReverAid First Strand cDNA Synthesis Kit (Vazyme Biotech Co. Ltd.). The relative transcriptional levels of silkworm *Sericin1*, *EGFP*, and *hEGF* were examined by quantitative real-time PCR (qRT-PCR) using SYBR Green Real-time PCR Master Mix (TOYOBO) with the following primers sets, *BmSer1RTF*: 5′- GGC​GAG​CTC​TAC​CAT​CTA​CG -3′ and *BmSer1RTR*: 5′- TCA​GAT​TTG​CTG​CGT​TTG​TC-3, *EGFPRTF:* 5′- GGT​GAA​CTT​CAA​GAT​CCG​CC-3′ and *EGFPRTR*:5′- CTT​GTA​CAG​CTC​GTC​CAT​GC-3′, and *hEGFRTF*: 5′- TGT​CCT​CTC​TCA​CAT​GAC​GG-3′ and *hEGFRTR*: 5′- ATG​ATG​GCG​TAA​TTC​CCA​CC-3’. The primer set that amplified a 136-bp fragment of *B. mori ribosomal protein 49* (*Bmrp49*) was used as the internal control (Li et al., 2018). Three independent biological replicates were used for all the qRT-PCR.

### Genotyping of G_1_ Animals

The G_1_ *DsRed2*-positive larvae were used for genomic DNA extraction. Insertion sites were confirmed using 5′- and 3′-end junction PCR using the primers as follows: *DsRed5′F*: 5′-CAG​AAG​TCA​TCG​TTC​AGG​CG-3′ and *DsRed5′R*: 5′-TCC​CAC​AAC​GAG​GAC​TAC​AC-3′ for 5′-junction PCR, *DsRed3′F*: 5′-CAG​TTC​GGT​TAT​GAG​CCG​TG-3′ and *DsRed3′R*: 5′-ATC​ACC​CAG​ACG​AAG​AGC​AA-3′ for 3′-junction PCR. Amplification products were cloned into the pJET1.0 cloning plasmid and sent for sequencing.

### Protein Extraction and SDS-PAGE Analysis

Silk proteins were extracted from the MSG of the wandering stage (W) larvae using the phosphate saline buffer (PBS) and silkworm cocoon shells were cut into small pieces for extraction using 8 M urea at 4°C overnight. The crude protein was quantified using BCA kit (Thermo) and sent for 10% SDS-PAGE. Separated proteins were treated with Coomassie brilliant blue (CBB) staining or transferred into the nitrocellulose membrane (GE Healthcare).

### Paraffin Embedding and Immunohistochemistry

Silkworm middle silk glands extracted from the *WT* or *Ser-2A-EGFP* animals were prefixed with Qurnah’s fixative. A 5-μm cross section was cut with a Leica RM2235 microtome and sent for staining according to our previous publication (Li et al., 2018). The sections were incubated with an anti-EGFP (1:2,000, ABclonal) primary antibody for 48 h and then washed for three times with PBS, followed by treatment with an FITC-conjugated goat-anti-rabbit secondary antibody (1:100, YEASEN). The nuclei were stained with Hoechst (1:1,000, Beyotime) for 10 min. Samples were analyzed with a fluorescence microscope (Olympus, BX53).

### Statistics Analysis of Data

All data were analyzed using GraphPad Prism (version 5.01) with two-way ANOVA and the Dunnett’s tests. The error bars are the means ± S.E.M. A *p-*value < 0.05 was used to determine significance in all cases.

## Results

### Targeting Silkworm *Sericin1* With Sequence-Specific Transcription Activator-Like Effector Nucleases

**FIGURE 1:**
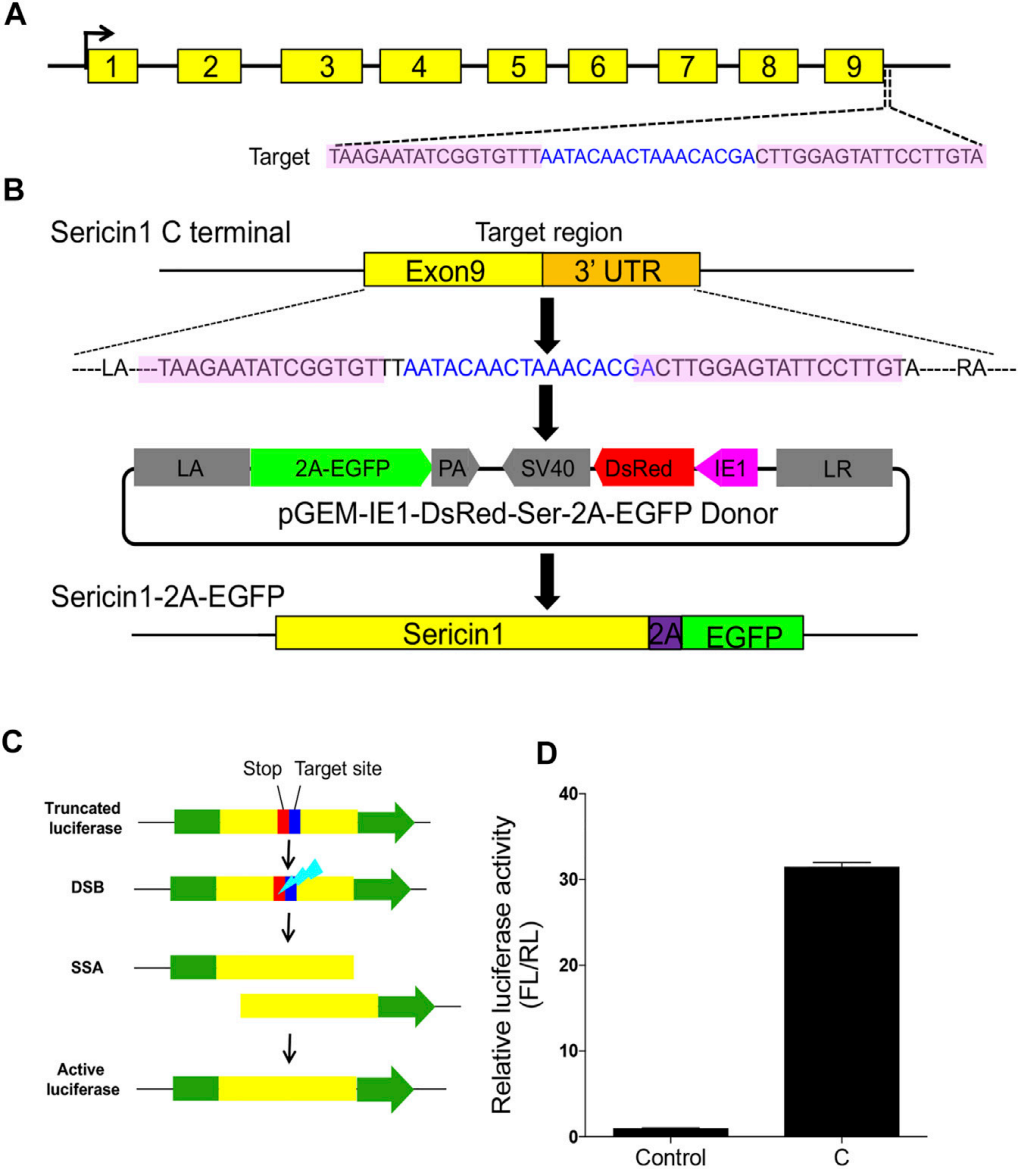
Designing and construction of TALENs. **(A)** Structure of silkworm *Sericin1* gene and the targeting sites of TALENs. The black arrows indicate the start codons. The black line is the genome DNA sequence and brown boxes represent *Sericin1* exons. Sequences of TALENs are also listed, with pink shaded letters indicating the TALEN recognition sites and blue letters are the spacers. **(B)** Scheme of 2A self-cleavage peptide (purple box)-mediated sericin-EGFP in-fusion expression on *Sericin1* loci (*Ser–2A–EGFP*). **(C)** Schema of SSA assay for TALEN cutting efficiency. Green boxes are firefly luciferase coding sequences, yellow boxes indicate the repeat fragment flanking the target site. The stop codon and target site are presented as red and blue boxes, respectively. **(D)** Luciferase activity of the TALENs used for integration. The bars stand for mean ± S.E.M (n = 3).

In the current study, we targeted the *Ser1* gene in *B. mori* to generate a *Ser1-EGFP* in-fusion expression transformant, as the proof-of-principle of our idea about the *in situ* expression of exogenous proteins in the silkworm silk gland. We used one pair of TALENs targeting sequence around the stop codons of *Ser1* to generate an in-fusion gene expression (Figure 1A). The donor template carried 1,000-bp length left and right homologous fragments which matched exactly to the sequences flanking the TALEN target, as well as the 2A self-cleavage peptide followed by the *EGFP* coding sequence and *Ser1* ployA sequences in between (*Ser-2A-EGFP*, Figure 1B). In theory, this scheme would express the target protein in the same manner with Ser1 since the common native regulatory elements were used. Given this idea, we designed three pairs of TALENs targeting the C-terminal of the *Ser1* gene, and selected the one with the highest cutting efficiency (31.5-fold to the control, Figure 1D), which was determined by an *in vitro* SSA assay to perform subsequent experiments (Figures 1C, D).

### Construction of *Ser1*-Targeted Transgenic Silkworms

**FIGURE 2:**
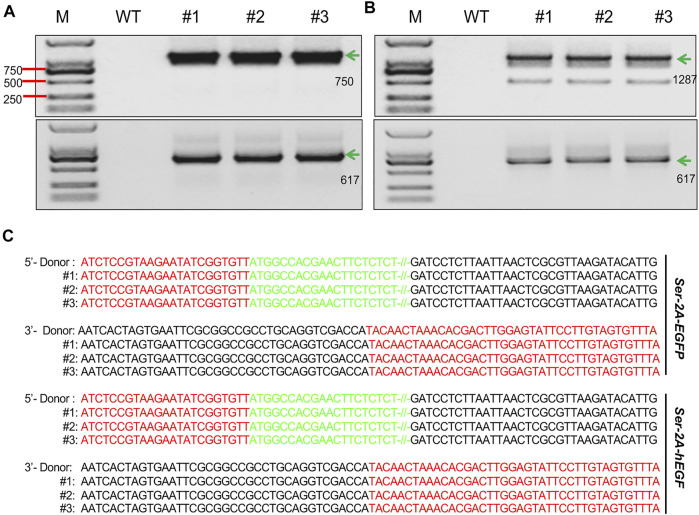
Genotyping of the transformed animals. **(A, B)** Junction PCR amplicons of the right and left flanking fragments in the *SKI–2A–EGFP* (A), and *Ser–2A–hEGF* (B) transformed silkworms. Three individual silkworms were used for detection. The green arrows indicate the correct amplicons, and their lengths are listed. M, marker; WT, wild type. **(C)** Sanger sequencing results for amplicons from **(B)** and **(C)**. Red letters stand for the sequences of homologous arms, green letters are the partial sequences of *EGFP* or *hEGF*, and black letters are the sequences of donor backbones.

*In vitro* synthesized TALENs mRNA and HR donors were coinjected into 640 silkworm preblastoderm eggs in each group to generate *Ser–2A–EGFP* transgenic line. In the G1 animals, five independent fluorescence-positive silkworm broods were obtained, achieving a 10.6% homologous recombination efficiency (Table 1). Genotype of the transformed animals were examined by through 5′- and 3′-junction PCR followed by Sanger sequencing using three animals from each G1 transformed silkworm broods. The results indicated that the integration events were precise and seamless (Figure 2A).

**TABLE 1:** Transformation efficiency of transcription activator-like effector nuclease (TALEN)-mediated transformation. Note. A total of 640 preblastoderm stage embryos were injected for each strain of animals.

Plasmid	No. of injections	G_1_ batch	Positive G_1_ batch	Transformation efficiency (%)
*Ser–2A–hEGF*	640	65	4	6.2
*Ser–2A–EGFP*	640	47	5	10.6

### Concordance of Enhanced Green Fluorescent Protein With Sericin1 Expression

**FIGURE 3:**
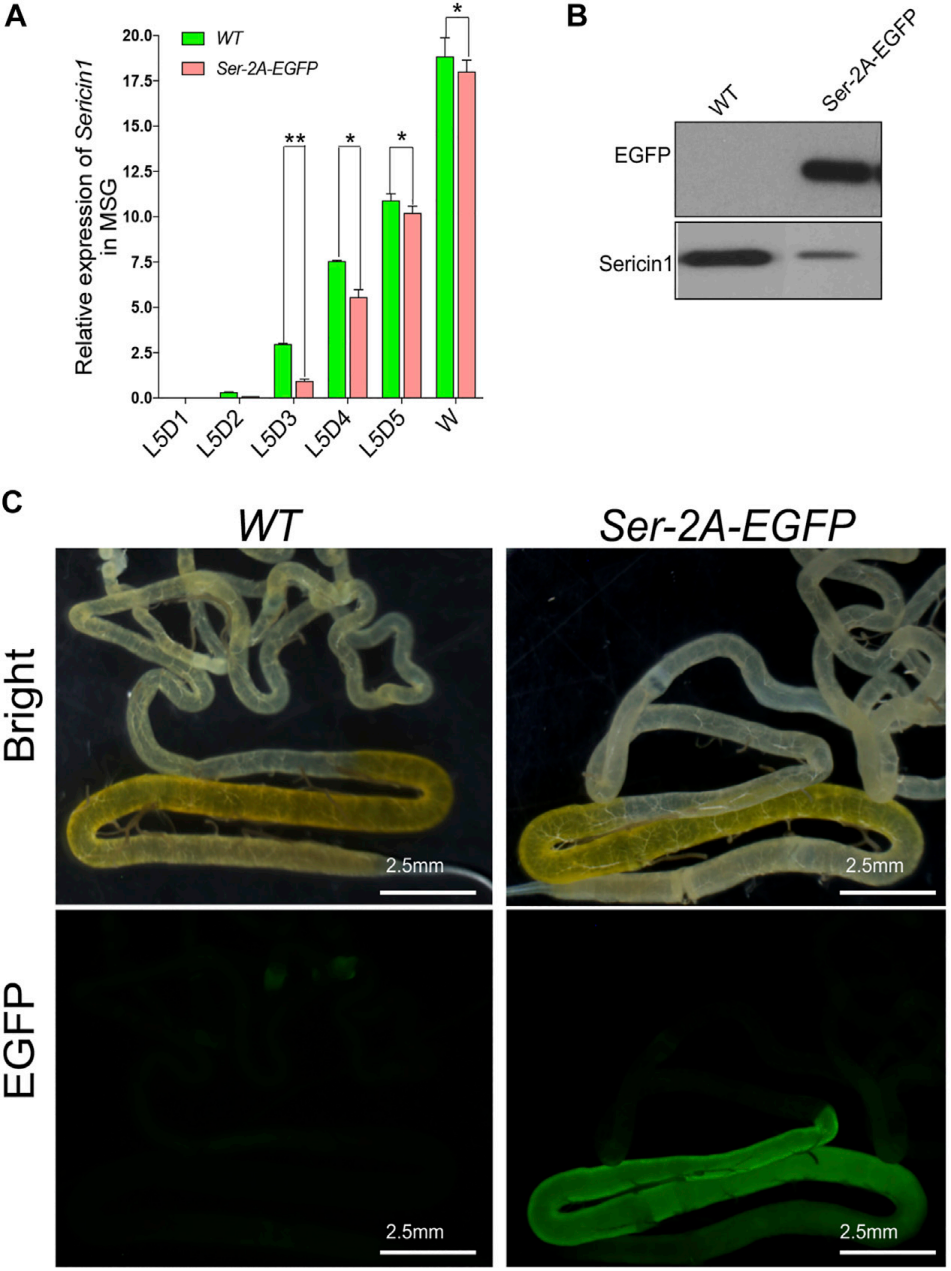
High production of enhanced green fluorescent protein (EGFP) protein by 2A mediated Sericin–EGFP in-fusion expression. **(A)** Relative transcriptional level of *Sericin1* in *WT* and *Ser–2A–EGFP* silkworms. qRT-PCR was used to quantify the transcriptional level of *Sericin1* and *EGFP* in **(A)** and **(B)**. The silkworm *ribosome protein 49* (*Bmrp49*) ortholog was used as the internal reference. Three individual replicates were used for qRT-PCR, and the error bars represent the mean ± S.E.M. **(B)** Western blotting of EGFP and Sericin1 protein in the middle silk gland (MSG) of wild-type (WT) and *Ser–2A–EGFP* silkworms at the wandering stage. **(C)** Fluorescence observation of the silk gland in the MSG of *WT* and *Ser–2A–EGFP* silkworms. Silk glands were extracted from the day 3 of the fifth instar larvae. Error bars stand for 2.5 mm.

In order to make sure that the EGFP insertion did not affect native *Ser1* gene expression, we first examined the transcription of *Ser1* by using qRT-PCR. In the heterozygous animals, *Ser1* presented relative low expression at the early stages of the final instar larvae, increased dramatically at the third day of larvae, and reached the peak at the wandering stage, being consistent with the WT animals (Figure 3A, Li et al., 2014). We observed that the expression level of *Ser1* in the *Ser–2A–EGFP* animals was significantly decreased at L5D3 and L5D4, being comparable with the wild type at the late larval stages (Figure 3A). This result indicated that the integration of *EGFP* coding sequence had some degree of impact to the native *Ser1* transcription (Figure 3A). We also detected EGFP proteins in the MSG of the *Ser–2A–EGFP* animals by Western blotting (Figure 3B). Observation of the bright EGFP fluorescence further confirmed the significant production of EGFP in *Ser–2A–EGFP* silkworm MSGs specifically (Figure 3C).

**FIGURE 4:**
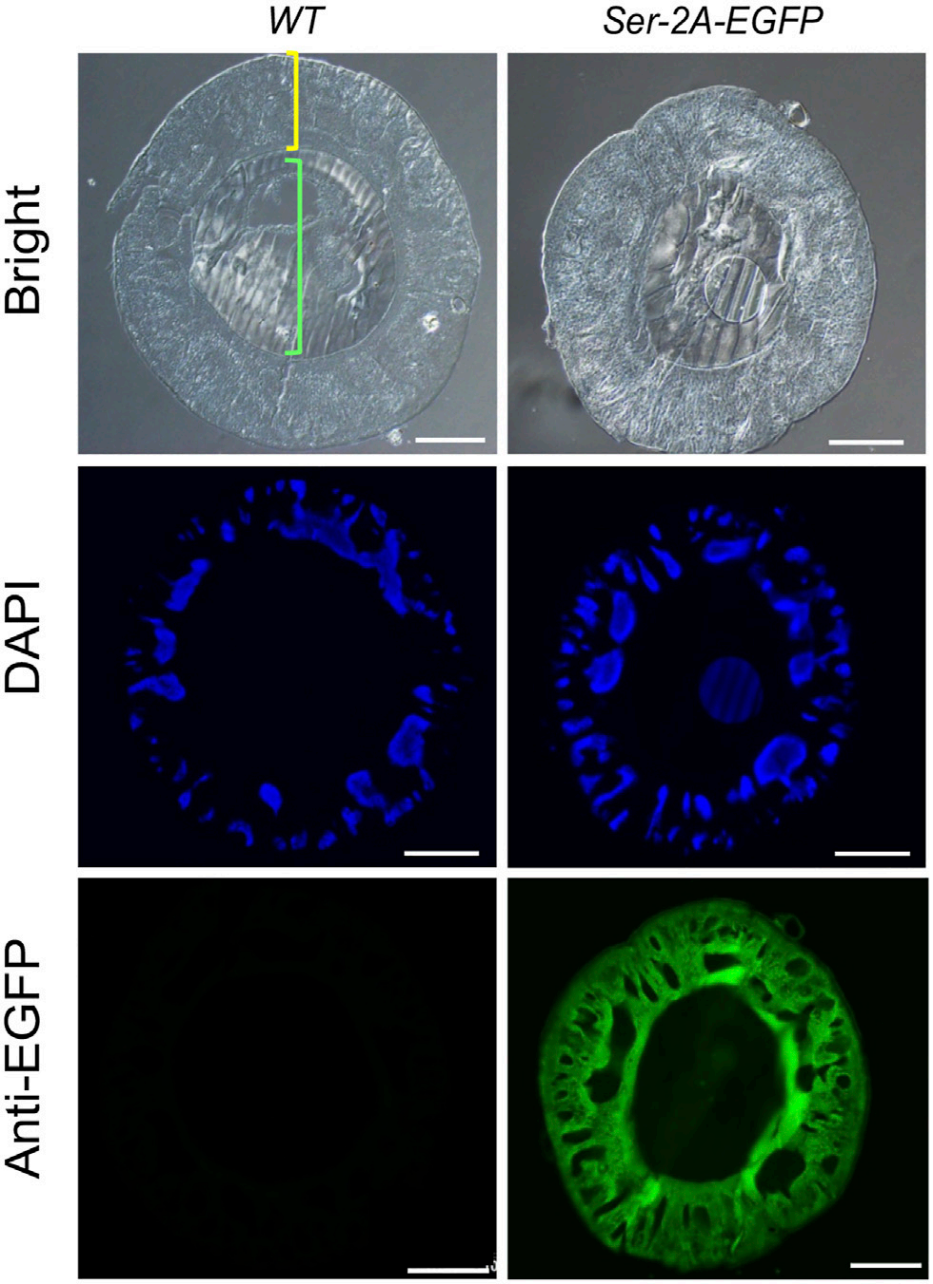
Immunohistochemistry of EGFP in the MSG of *Ser–2A–EGFP* animals. EGFP protein distributes in the sericin layer of the MSGs as indicated by the anti-EGFP primary antibody. The yellow bracket indicates the cellular layer and green bracket indicates the lumen of MSGs. MSGs were dissected from the *WT* or *Ser–2A–EGFP* animals at the third day of the fifth instar larvae (L5D3). Hoechst was used to stain the nucleus. Scale bars stand for 100 μm.

In the *Ser–2A–EGFP* silkworms, we assumed EGFP protein colocalized with endogenous Ser1 in the surface of the middle silk gland, since they shared the same regulatory elements. In fact, EGFP proteins were exclusively detected in the cell layer of MSGs by using anti-EGFP primary antibody at day 3 of the final larval instar, which coated the inner fibers constructed majorly with fibroin proteins (Figure 4). Furthermore, a cytoplasmic distribution of EGFP was detected by staining the nucleus with Hoechst. These results confirmed the exogenous EGFP protein expressed in the MSG efficiently and specifically.

### Expression of Enhanced Green Fluorescent Protein in the *Ser–2a–EGFP* Cocoon Shells

**FIGURE 5:**
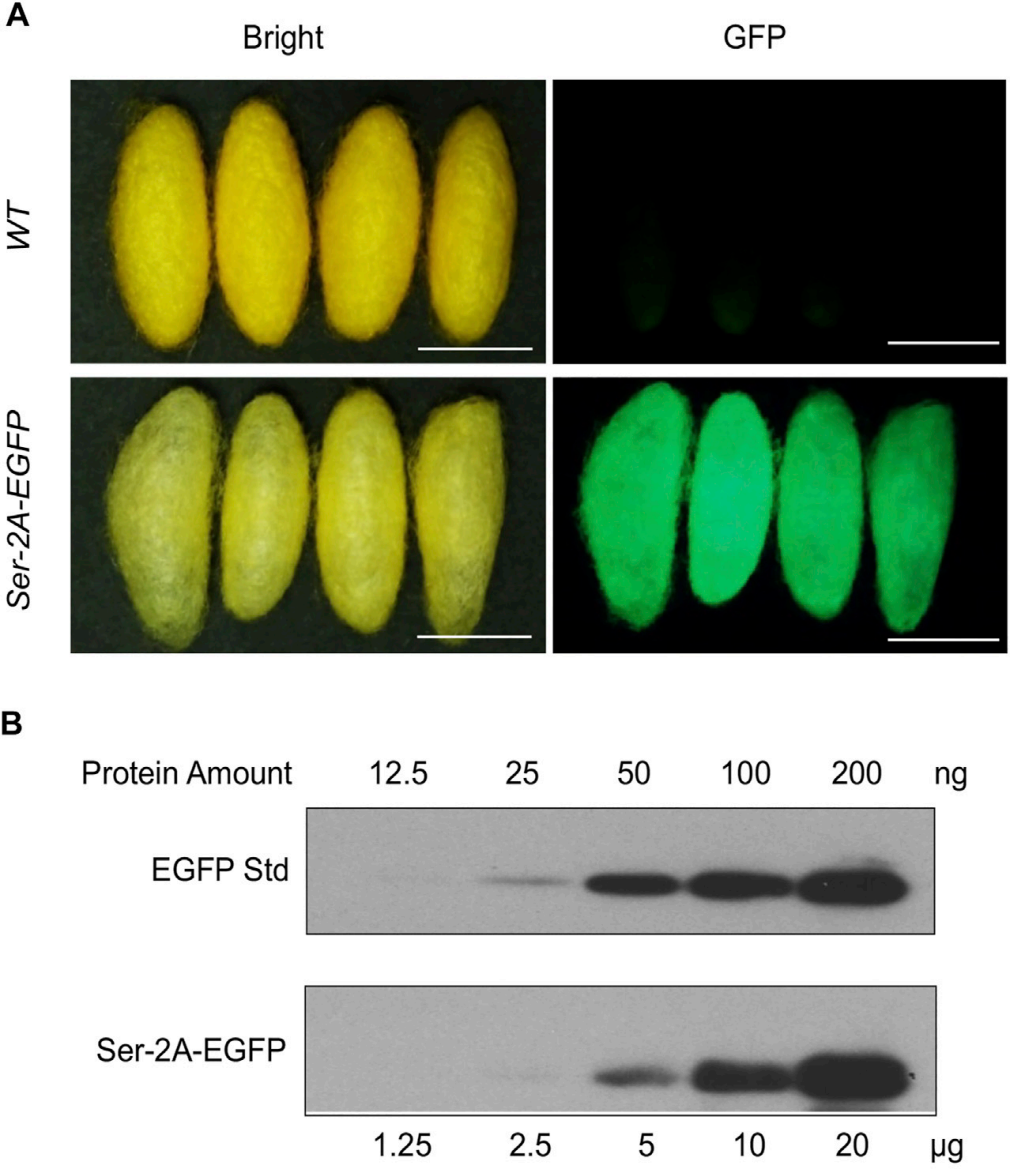
Quantification of EGFP protein in *Ser–2A–EGFP* cocoon shells. **(A)** Fluorescence observation of *Ser–2A–EGFP* cocoon shells. The upper row is the *WT* cocoon shells, and the lower row is the *Ser–2A–EGFP* cocoon shells. Both bright and GFP field are shown. Scale bars stand for 1 cm. **(B)** Quantification of the EGFP protein in the *Ser–2A–EGFP* cocoon shells. Proteins were diluted in a twofold series dilution manner and sent for analysis. The maximum amount for crude silk proteins and EGFP standard were 120 μg and 200 ng, respectively.

Being secreted together with Sericin1 protein, the EGFP protein was detected in the cocoon shells (Figure 5A). Nevertheless, the chimeric cocoon shells of *Ser–2A–EGFP* heterozygous animals were thinner and softer than the WT as we observed (Figure 5A). We measured the average weight of *Ser–2A–EGFP* silkworm cocoon shell, and found that it was decreased to 90.7% (0.107 ± 0.008 g) and 81.4% (0.092 ± 0.010 g) of the WT females and males, respectively (Table 2).

**TABLE 2:** Comparison of the silkworm economic traits.

Genotype	Female			Male		
	Whole cocoon weight (g)	Cocoon shell weight (g)	Cocoon shell rate (%)	Whole cocoon weight (g)	Cocoon shell weight (g)	Cocoon shell rate (%)
*WT*	1.127 ± 0.046	0.118 ± 0.008	10.45 ± 0.003	0.890 ± 0.030	0.113 ± 0.011	12.65 ± 0.005
*Ser-2A-hEGF*	1.062 ± 0.049^a^	0.105 ± 0.007^a^	9.91 ± 0.004^a^	0.825 ± 0.047^a^	0.097 ± 0.011^a^	11.66 ± 0.007^a^
*Ser-T-hEGF*	1.023 ± 0.047^a^	0.107 ± 0.012^a^	10.42 ± 0.011^a^	0.754 ± 0.046^a^	0.097 ± 0.013^a^	12.95 ± 0.016^a^
*Ser-2A-EGFP*	0.913 ± 0.038^a^	0.107 ± 0.008^a^	10.17 ± 0.005^a^	0.766 ± 0.041^a^	0.092 ± 0.010^a^	12.49 ± 0.006^a^

Note. Female and male animals were separated and used for statistics. The data shown are mean ± S.D. (n = 30). The asterisks stand for significance with *p < 0.05*.

a Significance between WT and transformed lines.

To quantify the production of EGFP protein in the cocoon shells of *Ser–2A–EGFP* animals, we extracted the crude proteins from the cocoon shells by using 8 M urea at 4°C overnight and performed Western blotting analysis. The experiment was conducted using both the extracted crude proteins and EGFP standard protein with a twofold series dilution (Figure 5B). In the heterozygous animals, the yield of EGFP protein reached to 1.05% (w/w) of the cocoon shell weight, which was higher than the existing transgenic-mediated production as reported previously (Figure 5B).

### Expression of the Short Peptide *hEGF* Using *Sericin1* In-Fusion System

Since silkworm sericin protein is hydrophilic and does not cause allergic reactions, it has been widely used as a new medical material, including in wound healing (Aramwit et al., 2012). Here, we applied our sericin in-fusion expression system to produce the hEGF protein, the substrate of EGFR signaling which involves in re-epithelialization of epidermal wound healing and keratinocyte stem cell proliferation, in silkworm cocoons (Nanba et al., 2013). The *hEGF* gene was 183 bp and encoded a 7.2 KD short peptide. Here, we injected 640 WT eggs with the mixture of TALEN mRNA and donor plasmid, and four fluorescence-positive silkworm broods were obtained from a total of 65 G1 broods, the transformation efficiency reached 6.2% (Table 1). At the same time, transposon-based, transgenic silkworm expressing the *hEGF* driven by *Ser1* promoter (*Ser–T–hEGF*) was also constructed as the control to compare protein yields with the *Ser–2A–hEGF* animals.

**FIGURE 6:**
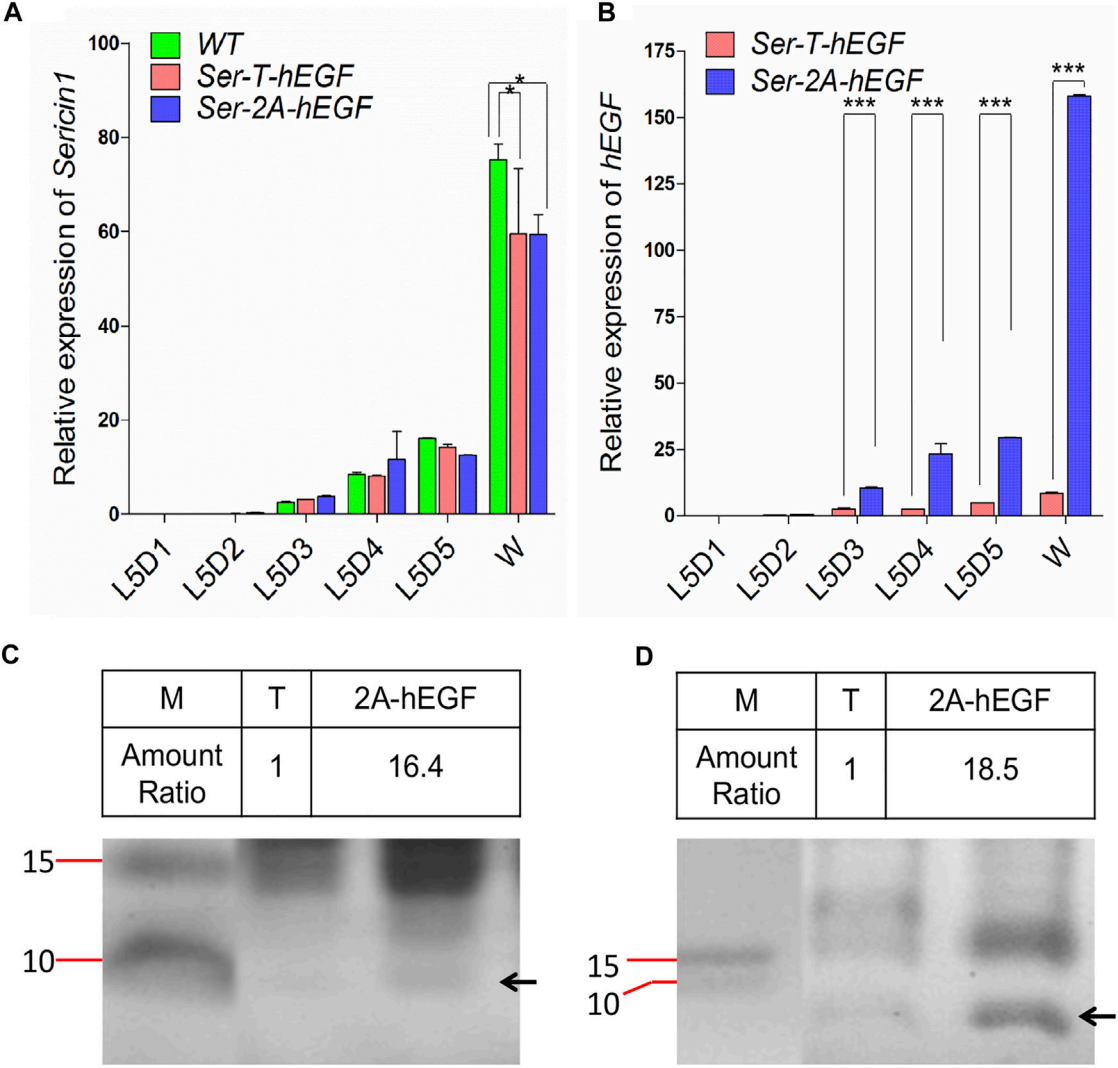
Expression of human epidermal growth factor (hEGF) protein in the *Ser–2A–hEGF* animals. **(A)** Comparison of *hEGF* transcriptional levels between *Ser–T–hEGF* and *Ser–2A–hEGF* animals. **(B)** Detection of *Sericin1* in *WT*, *Ser–T–hEGF*, and *Ser–2A–hEGF* silkworms. qRT-PCR was used to quantify the transcriptional levels of *Sericin1* and *hEGF* in **A** and **B**. The silkworm *ribosome protein 49* (*Bmrp49*) ortholog was used as the internal reference gene. Three individual replicates were used for qRT-PCR, and the error bars represent the mean ± S.E.M. **(C, D)** Coomassie brilliant blue staining for the proteins extracted from the MSG (D) and cocoon (D) of *Ser–T–hEGF* and *Ser–2A–hEGF* silkworms. The black arrows indicate hEGF protein. M, protein marker; T, *Ser–T–hEGF*; 2A-hEGF, *Ser–2A–hEGF*. Size of protein marker bands are listed.

We first examined the expression level of *Sericin1* gene, and no significant difference was observed when comparing both transformed lines to the WT silkworms (Figure 6A). However, the transcriptional level of *hEGF* was increased to 18-fold of that in *Ser-T-hEGF* animals at the W stage (Figure 6B), suggesting the native *Ser1* regulatory elements or the local genomic DNA context were also important for target gene expression. Significant increase on the hEGF protein expression was detected in both the MSG and cocoon of *Ser–2A–hEGF* when compared with the *Ser–T–hEGF* animals, respectively (Figures 6C, D). Altogether, we successfully adapted the *Ser1* in-fusion system to express the therapeutically important factor hEGF, which is promising for being used as a new biomedical substrate.

## Discussion

In the current study, we successfully established a *Ser1* in-fusion expression system by using the TALEN-mediated, targeted gene integration in *B. mori*. This strategy was then used to express recombinant proteins specifically in the middle silk gland driven by the natural *Ser1* regulatory elements.

The silkworm silk gland has been developed as an efficient bioreactor for a long period based on transposon-based transgenesis (Royer et al., 2005; Mori et al., 2014). Two systems, fibroin and sericin, have been developed for recombinant protein expression (Zhao et al., 2010; Wang et al., 2015; Xu et al., 2018). However, transposon-based transgenic production of the exogenous proteins was limited for several reasons. First, *piggyBac*-mediated transgene introduces the exogenous fragments into the undefined genome locus. Therefore, the genetic background and production of recombinant protein varied largely between different transgenic lines (Tomita et al., 2007; Wang et al., 2013). In addition, transposon-mediated transgene is instable and often causes gene drift. Besides, integrated fragments introduced by transgenesis are out of strict control and ubiquitously expressed, causing toxicity for the host and leading to high mortality (Tomita et al., 2007).

Here we performed a 2A-mediated *Ser1* in-fusion expression by creating double-stranded breaks at the C-terminal of the *Ser1* genomic loci, and integrated either EGFP or hEGF at the downstream seamlessly. High production of EGFP was detected in *Ser–2A–EGFP* animals in both the middle silk glands and cocoon shells. It was attributed to this system that uses the whole *Ser1* promoter, which may optimize the promoter activity. Another benefit is that the in-fusion expression system did not disrupt the expression of the natural *Ser1* gene and has less effect on that according to our results, loading few fitness costs on the host animals compared with the transgene strategy. However, we still observed that the overexpression of *EGFP* with this strategy produced a thinner cocoon shell (Figure 5A), which had the similar phenotype with the fibroin-deficient line *Nd-s* ^*D*^, implying that the native *Fibroin* gene expression may be affected, which mechanism needs further exploration. In regard to the comparison between *Ser–2A–EGFP* and *Ser–2A–hEGF* lines, we also observed some degree of effect on the *Ser1* expression with the smaller peptide (hEGF) integration (Figure 6A), we assumed that was caused by the toxicity by the high expression of hEGF in the MSG. It also should be noticed that the protein size-dependent effect on the fitness cost, since larger proteins being fused with sericin, the higher the possibility for deformed configuration on Sericin1 protein itself. Overall, this in-fusion expression strategy holds the great potential for recombinant protein expression in the silk gland.

In addition, the 2A-mediated in-fusion expression for hEGF increased to more than 15-fold than that in the transgenesis animals (*Ser–T–hEGF*, Figure 5A). In *Ser–2A–hEGF* animals, the completely native promoter and other upstream regulatory elements of *Ser1* gene were subjected, rather than only the seed sequence of the promoter was used in the *Ser–T–hEGF* animals, which excludes the possibility that some potential enhancers existed in the upstream of the *Sericin1* promoter. We also cloned a 404-bp 3′ UTR sequences of *Ser1* and inserted it into the downstream of *EGFP* or *hEGF*, further mimicking the native *Ser1* expression. Furthermore, we used the *Thosea asigna* virus-derived T2A self-cleavage peptides to achieve the in-fusion expression (Diao and White, 2012). T2A peptide forces the ribosome skip between the glycine and proline amino acids, without the peptide bond during translation, therefore the native *Ser1* and linked EGFP or hEGF are transcribed together with Sericin1 but translated independently.

Actually, the *Ser–2A* system has wide usage on both genetics and biochemistry other than the application for single protein expression. One example is expressing tandem linkage of more than one copy of hEGF or other proteins in MSG, which may further increase the expression efficiency. In addition, 2A peptide can be used to link multiple protein-coding sequences tandemly and to be controlled by a single promoter, simplifying the construction aim to expression multiple factors. In addition to in-fusion with sericin, the exogenous proteins or sequences can be inserted into any site desired including the exon, intron, and even the untranslated regions, reducing the side effect on the targeted gene itself, and it also can be used for protein tagging or manipulation. In conclusion, the current work established a natural *Ser1* expression system, providing us a new genetic strategy for the mass production of exogenous proteins and further promote the silk gland to be an excellent bioreactor system.

## Data Availability

The original contributions presented in the study are included in the article/Supplementary Material, Further inquiries can be directed to the corresponding author.
